# Ways forward for Machine Learning to make useful global environmental datasets from legacy observations and measurements

**DOI:** 10.1038/s41467-022-32693-3

**Published:** 2022-09-07

## Abstract

Advances in geospatial and Machine Learning techniques for large datasets of georeferenced observations have made it possible to produce model-based global maps of ecological and environmental variables. However, the implementation of existing scientific methods (especially Machine Learning models) to produce accurate global maps is often complex. *Tomislav Hengl* (co-founder of OpenGeoHub foundation), *Johan van den Hoogen* (researcher at ETH Zürich), and *Devin Routh* (Science IT Consultant at the University of Zürich) shared with *Nature Communications* their perspectives for creators and users of these maps, focusing on the key challenges in producing global environmental geospatial datasets to achieve significant impacts.

Tell us about your background and how you combine geospatial mapping with data in your work?

**TH (Tom):** My background is in environmetrics and data science. I have a BSc in Forestry, MSc in Geoinformation science, and PhD in Pedometric mapping from Wageningen University & ITC. I am currently a director at the OpenGeoHub foundation. From the beginning of my science career, I have been fascinated with detecting and understanding spatial patterns in nature and using that knowledge to accelerate land restoration/regreening of the planet. I work with global maps and spatial analysis on a daily basis. I currently lead two EU projects that focus on producing frameworks for automated mapping and monitoring: Open Data Scienc and Open Earth Monitor. I also lead summer schools and hackathons for the MOOD project.

**Figure:**
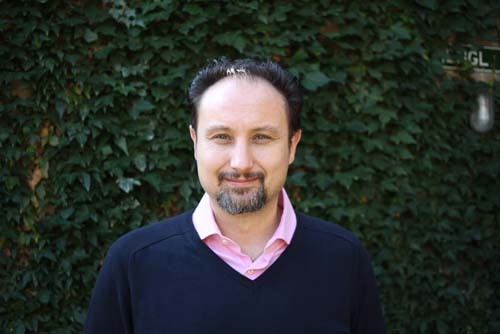
Dr. Tomislav Hengl, Director, and founder of OpenGeoHub Foundation. Tom Hengl

**JVDH (Johan):** I’ve always been fascinated by nature, especially how in natural systems even the smallest things can have very large effects. During my PhD in Molecular Biology, I studied the functioning of catalytic domains in cell surface receptors of plant pathogens. In my current position as research scientist at ETH Zürich, I try to understand how microbial life contributes to the global carbon cycle. In this global-scale approach, geospatial maps are indispensable, as they can help us to identify regions that might be at risk with global change, or which are currently underrepresented in global datasets.

**Figure:**
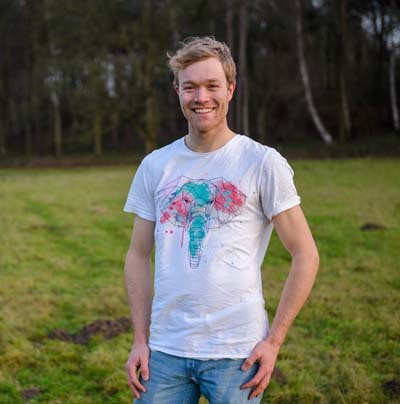
Dr. Johan van den Hogen, research scientist, Global Ecosystem Ecology, ETH Zürich. Joris Schaap

**DR (Devin):** Growing up on my family’s farm around land managers and farmers spurred me to study forest science. Both GIS and remote sensing—and thus mapmaking via the use of geospatial data—are valuable assets for foresters and other land managers. As a scientist, I have applied and continue to apply skills with spatial data in research that ranges from global ecosystem ecology to land surface phenology. I currently work as a Science IT Consultant at the University of Zürich (Switzerland), and I contribute to the GlobDiversity project as well as the Swiss NPOC alongside my colleagues at the RSL.

**Figure:**
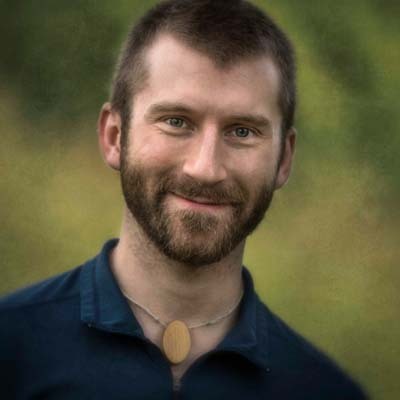
Devin Routh, Science IT Consultant, Universität Zürich. Dan Routh

What are the main technical challenges in producing global environmental datasets?

We can group the current main challenges in producing global environmental datasets into five categories: (i) training data issues—these include mismatches in standards when recording measurements, legal and technical issues that limit data use, high costs of producing new observational data; (ii) modeling issues—these include point clustering and extrapolation problems, model overfitting, artifacts in input data, lack of historical data, lack of consistent global monitoring stations^1^, limited availability and/or quality of global covariate layers; (iii) data distribution issues—these include unusable file formats, high data volumes, incomplete or inaccurate metadata; (iv) usability issues—these include incompleteness of data, unawareness of user communities and/or data limitations, data being irrelevant for decision making; and (v) governance issues—these include closed data policies from (non-)governmental organizations, datasets not used by organizations, lack of baseline estimates, absence of strategies for updates of data products. Considering the urgency of the global biodiversity and climate change crises, we do not have the luxury to wait until scientists solve all these challenges before making global datasets for subsequent use and distribution. As policymakers need ever more up-to-date information to make the most informed decisions possible^2^, global-scale, wall-to-wall, models and maps are indispensable and can provide significant added value compared to local models^3^. Consequently, it is absolutely critical that global data products are accompanied by the appropriate metadata information and spatial information of uncertainty and prediction errors.

You mention extrapolation as one of the main modeling challenges today. When should we stop extrapolating in data science and how can we limit the risks of extrapolation?

There has been some criticism recently directed at global models that extrapolate predictions into areas that lack adequate training data (e.g., ref. 4). This is especially true when global models, fit using Machine Learning methods, are used to predict outside the geographic or temporal limits of the training space, including into unsampled geographic regions or far into the future. We assert that extrapolation is, in fact, often very useful and should not be discouraged or considered a fatal flaw in the modeling process. As an example, consider global climate scenario modeling: though we can never perfectly predict and validate what the climate will be in 50 years, or what the potential optimum of natural ecosystems is, we should embrace credible, i.e., statistically rigorous, climate extrapolation exercises (e.g., AR6 climate scenarios and maps of potential natural vegetation).

To ensure that future mapmakers have specific guidance on how to release their data products responsibly, we propose that whenever possible mapmakers release at least two accompanied data layers for each predicted layer that is created: (1) a layer showing the uncertainty of predictions per pixel value (i.e., an error distribution of the predicted value); (2) a layer showing the degree to which the training data matches the area being predicted (i.e., a layer highlighting the degree of extrapolation, or similar). Moreover, the modeling process itself should make the best use of uncertainty estimates; a technical example could be bootstrapping training data so that model training datasets use the provided error distributions sampled at the per-pixel level. If training datasets don’t themselves contain error uncertainty values, scientists could experiment with Bayesian-style priors that they themselves estimate so that uncertainty can still be properly represented and propagated (e.g., ref. 5).

Deriving local prediction errors for complex machine learning models are, however, computationally demanding and data producers often need to use non-parametric bootstrapping methods^6^. These can significantly increase production costs, ultimately leading to delays and/or to a reduction in the number of mapped variables. In one of our recent analyses^7^, the prediction error maps required an order of magnitude more computational effort than the actual predictions. Overall, the ultimate goal of mapping should be to present the pixel level information at every point in addition to a full suite of accuracy statistics (output probability distributions) at every point or at least for the whole area of interest, which requires a great degree of planning, computational power, storage space, and fully transparent communication in results.

In summary, producing global consistent and usable datasets takes time, effort, and healthy scientific debate. In the context of inevitably limited and imperfect datasets, more information on error estimations is superior to less information (i.e., masking pixels). Extrapolation inevitably comes with risks, but it is always useful to test scenarios so that the scientific community can advance our work and continuously improve our methods.

Can you elaborate on some of the other main technical challenges and the approaches you adopt to address them?

The ever-present and key technical challenges to our work can be roughly categorized as: (1) large data volumes, (2) significant time and effort required to find, clean, and improve reliable training data, (3) difficulty in increasing the usability of data we produce without making significant additional investments. We typically address many of these problems by becoming more efficient coders, but sometimes even profound coding skills can’t save you! In these cases, the most obvious solutions are to either partner with experienced data scientists and/or to obtain more computing power. In the case of global space/time modeling and mapping, there is the additional challenge that one cannot travel to the past or the future, or even necessarily across the globe, to collect more training data or to validate global spatiotemporal models.

Overfitting is also a serious problem in data science. An effective strategy to avoid overfitting is to spend more effort testing model performance using various resampling strategies^6^ and then see how the model performs, especially if a realistically simulated probability re-sampling is used. We recommend that, as often as possible, scientists fit their models using Ensemble Machine Learning frameworks such as mlr3, h2o, or scikit-learn that come with robust mechanisms to reduce overfitting, via meta-learners or stacking of the models.

Another serious challenge is the problem of artifacts in input data. It is well known that many Machine Learning algorithms are sensitive to data artifacts. Algorithms such as Random Forest may be noise proof^8^, yet they are sensitive to artifacts and bias in the training data. For example, some data producers use 0 for missing values; when not properly cleaned or accounted for, even a very small portion of such “polluted” data can have an enormous impact on the results. Luckily there are more and more diagnostic tools to visualize and investigate data quickly (especially multivariate correlation plots and density plots) to identify artifacts. The R and python open source development societies provide many such tools that are available completely open source (e.g., ref. 9). It is crucial to fully explore the data before production runs to ensure datasets have been satisfactorily cleaned and to document the methodology to ensure end-users can both understand and reproduce your work.

What is your advice for researchers starting to work with global maps?

Researchers looking to begin working with global spatial datasets should start by creating a tabular overview of relevant and available data sources that lists each dataset’s technical specifications (e.g., title, source, target variable, reference measurement & classification method, spatial resolution, temporal coverage, completeness, consistency, versioning, etc.). This overview can be used to decide whether to use a single dataset or a combination of datasets. Of course, it is important to remember that no map is perfect. A good practice here is to check whether the map is accompanied by a prediction error or accuracy map, what its limitations are, and whether there have been updates issued. Remember to be cautious if the area of interest is outside the training space of the original model!

What role can open science play in increasing accessibility of global environmental data?

Open Science is a remedy for many of the challenges we listed previously, especially through the practice of reproducible research. A key task for any modern researcher is to fully document their code and workflows in a reproducible and accessible method without restrictions. Open science, specifically the registration of data and code on professional repositories using open source licenses and open data licenses, enables other groups to easily find and access data, validate results, and to build upon, as well as improve, the process. To achieve openness, dataset metadata should also be provided following FAIR principles. With open data and code repositories such as Zenodo.org, GitHub.com and/or GitLab.org, researchers have the capacity to register both data and code that facilitates other interested parties to become involved. Going further, all parts of a scientific workflow can be documented on platforms such as https://osf.io and/or https://www.octopus.ac. Of course mistakes will inevitably exist in research code, and these tools can be used to regularly find and address them. To open your research you should first consider documenting your work through computational notebooks and tools such as R bookdown, Jupyter Notebooks and similar tools. Furthermore, there are plenty of tools today to expose your data science problems (e.g., through platforms such as OpenML and Kaggle) so that other data scientists can share their creativity and jointly solve world crises. Scientists should strive to cultivate a culture that celebrates finding and correcting mistakes without shame or judgment so that the entire scientific community can progress.

Do you have any advice for scientists who are not geospatial experts but are interested in using geospatial data and maps?

A good first introduction for non-geospatial experts to start exploring maps is to use popular data portals where important maps are available. Some useful web-mapping portals are Global Forest Watch for monitoring global forests, the Copernicus Mapping Portal for monitoring NOx and CO2 emissions, and Google's Timelapse (powered by Google Earth Engine) for visually observing changes in environments over long periods of time. For those users who would like to get deeper into using maps or at least customizing how to visualize maps, we recommend downloading and installing the free and open-source GIS QGIS, then opening and browsing maps of interest together with related vector and/or background layers.

*This interview was conducted by Walter Andriuzzi*.
